# fNIRS estimates prefrontal cortex activation following glucose load in youths with obesity

**DOI:** 10.1007/s40618-026-02911-6

**Published:** 2026-05-26

**Authors:** Gabriele Scozia, Deny Menghini, Stefano Lasaponara, Alessia Aureli, Chiara Carducci, Valentina Quaresima, Roberto Isabella, Fabrizio Doricchi, Stefano Vicari, Stefano Cianfarani, Danilo Fintini, Melania Manco

**Affiliations:** 1 Child and Adolescent Neuropsychiatry Unit, Department of Neurological and Psychiatric Science, Bambino Gesù Children’s Hospital, IRCCS, Rome, Italy; 2 Unit of Preventive and Predictive Medicine, Bambino Gesù Children’s Hospital, IRCCS, Via F. Baldelli 38, 00146 Rome, Italy; 3 Dipartimento di Psicologia, Università degli Studi di Roma ‘La Sapienza’, Rome, Italy; 4 Fondazione Santa Lucia IRCCS, Rome, Italy; 5 Endocrinology and Diabetes Unit, Bambino Gesù Children’s Hospital, IRCCS, Rome, Italy; 6 Dipartimento di Medicina Clinica, Sanità Pubblica, Scienze della Vita e dell’Ambiente, Università degli Studi dell’Aquila, L’Aquila, Italy; 7 KeyRIB for ANUA E.T.S. Association - Social Enterprise, Rome, Italy; 8 Department of Life Science and Public Health, Università Cattolica Del Sacro Cuore, Rome, Italy; 9 Department of Systems Medicine, University of Rome Tor Vergata, Rome, Italy; 10 Department of Women’s and Children’s Health, Karolinska Institutet, Stockholm, Sweden

**Keywords:** Children, Insulin sensitivity, Dorsolateral prefrontal cortex, Functional near-infrared spectroscopy, Obesity

## Abstract

**Purpose:**

The prefrontal cortex [PFC] is crucial in controlling food-related impulses. Functional near-infrared spectroscopy [fNIRS] represents a wearable functional neuroimaging technology for measuring PFC activation in real life. Aims of the study were to examine PFC activation in fasting conditions and after an oral glucose tolerance test [OGTT] in resting- state [R], cognitive tasks [Ts] conditions and explore the PFC activation relationship with insulin sensitivity index [ISI] in youths with obesity.

**Methods:**

28 patients’ cross-sectional study [age 7—15 y]. ISI was quantified by Matsuda’s Index and PFC activation as oxygenated [O_2_Hb, mol/L] and total [tHb, mol/L] hemoglobin concentration changes, in fasting and during the first 60 min of the OGTT; in resting-state condition and during cognitive tasks [Ts: Verbal Fluency, Digit Span, Non-Verbal Stroop] administered before [Fasting-Ts], and 15 min after OGTT [OGTT-Ts]. 15 min OGTT-R was divided into 5 time periods [P1-P2-P3-P4-P5], and cognitive tasks [OGTT-Ts] were then administered again.

**Results:**

Dorsolateral PFC [DLPFC] and right frontal pole were over-activated in OGTT compared to the fasting state. Significant differences [p < 0.005, false discovery rate corrected] were observed: P2 [O_2_Hb Left DLPFC Fasting-R: 1.47_*_10^–6^ vs OGTT-R: 4.13_*_10^–6^ mol/L], P3 [tHb Right DLPFC Fasting-R: 1.15_*_10^–6^ vs OGTT-R: 3.36_*_10^–6^ mol/L], and P5 [ O_2_Hb Left DLPFC Fasting-R: 1.47_*_10^–6^ vs OGTT-R: 4.34_*_10^–6^ mol/L, tHb Right DLPFC Fasting-R: 1.15_*_10^–6^ vs OGTT-R: 3.07_*_10^–6^ mol/L]. The change of left DLPFC activity correlated significantly with ISI [r = 0.61, p < 0.001].

**Conclusion:**

fNIRS enabled the non-invasive examination of the PFC hemodynamic response associated with participants’ insulin sensitivity in an ecological context. Bilateral DLPFC activation following OGTT in youths with obesity was associated with peripheral ISI. This association suggests that peripheral insulin sensitivity might reflect the PFC’s ability to control food-related behaviours.

**Supplementary Information:**

The online version contains supplementary material available at 10.1007/s40618-026-02911-6.

## Introduction

Worldwide, 340 million children and adolescents aged 5—19 live with overweight or obesity. Obesity in youth is associated with higher circulating insulin levels and insulin resistance, conditions that are exacerbated at the pubertal transition [1]. Insulin plays a significant role in cerebral bioenergetics, promotes synapse viability and dendritic spine formation, and boosts the turnover of neurotransmitter, such as dopamine that plays a fundamental role in the eating behaviour of "wanting" via the reward system [2–4]. Additionally, insulin regulates vascular function by influencing vasoreactivity, lipid metabolism, and inflammation [5].

Neuroimaging studies have shown that insulin significantly modulates brain activation, with all brain regions responsive to the hormone [6, 7] including. The prefrontal cortex [PFC] and in its sub regions, i.e. the Orbito Frontal Cortex [OFC], the Ventral Lateral PFC [VLPFC], and the Dorsolateral Prefrontal Cortex [DLPFC], that are key regions in executive function and behavioral self-regulation [4, 8, 9]. Disruption in executive function can lead to poor dietary choices and overeating [12]. Indeed, the DLPFC is differently activated in adults and children with obesity compared to the DLPFC of normal-weight individuals [10, 11] and children with obesity generally score lower on measures of executive function, including working memory, reward-sensitivity, and inhibitory control [13–15].

Following glucose intake or a meal in healthy hungry subjects, neural activity (revealed as regional cerebral blood flow by positron emission tomography) decreases in limbic/paralimbic areas whilst it increases in the PFC that is particularly sensitive to increasing peripheral and central insulin levels [8].

Youths with obesity may exhibit impaired glucose metabolism in the PFC that, in turn, could influence responses to certain cognitive tasks. Existing studies have primarily employed functional magnetic resonance imaging [fMRI] or positron emission tomography and have mostly focused on adults [16, 17].

In an fMRI study, Jastreboff et al. [18] found decreased perfusion in the PFC following the OGTT in adolescents with obesity, compared with lean peers, who, conversely, showed increased perfusion correlated with insulin sensitivity.

Functional near-infrared spectroscopy [fNIRS] is a non-invasive optical neuroimaging technique that assesses cortical hemodynamics via neurovascular coupling, inferring neural activity from changes in oxygenation [deoxygenated-hemoglobin: HHb; oxygenated-hemogobin: O_2_Hb, total hemoglobin: tHb(O_2_Hb + HHb)] of the cerebral microcirculation blood vessels [19, 20]. The coupling between neuronal activity and cerebral blood flow is at the basis of brain function, and fNIRS relies on this coupling to infer changes in neural activity, which is reflected by the blood oxygenation changes of the activated cortical region (i.e., the increase in O_2_Hb and the concomitant decrease in HHb). fNIRS was validated against fMRI blood-oxygen-level-dependent responses [21].

fNIRS wearability in ecological settings makes it ideal for studying brain function in youths [22]. The use of fNIRS has led to advances across fields, such as cognitive neuroscience, clinical applications, developmental science, and sport and exercise science, among others.

To date, no studies have combined fNIRS with measures of insulin sensitivity in pediatric populations and, in general, the interaction among insulin sensitivity, cognitive performance, and PFC hemodynamics in youth remains largely unexplored.

The present pilot study aimed to investigate PFC activation using fNIRS, in children and adolescents with obesity, in relation to insulin sensitivity and cognitive performance, e.g. in fasting conditions and after OGTT, in resting conditions and during neuropsychological tasks.

We aimed also at exploring the feasibility and applicability of fNIRS as a neuroimaging tool in terms of technical implementation, children’s compliance and data quality, to quantify metabolism-related PFC activity.

## Methods

### Participants

The study enrolled 28 consecutive outpatients with overweight referred to the "Endocrinology and Diabetology" unit of the Bambino Gesú Children’s Hospital [Italy] from June 2022 to August 2024.

Participants were asked to refrain from intensive physical activity for 3 days prior to the study and to have a standard dinner the day before the study.

After a 12-h overnight fast, children were admitted to the clinic for a 1-day inpatient visit to undergo an OGTT, fNIRS and cognitive tasks.

*Inclusion criteria*: age ≥ 7 and < 15 years, right-handed, overweight or with obesity, Body Mass Index (BMI) ≥ 85th percentile (1.036 SDS) and < 95th percentile (1.645 SDS), or ≥ 95th percentile, according to Italian growth charts [23].

*Exclusion criteria:* inability to provide informed assent to the study, genetic or syndromic obesity, systemic, neurological, or psychiatric conditions or any other condition associated with a systemic inflammation, chronic use of medications; non-verbal intellectual quotient ≤ 75 as measured by Colored Progressive Matrices [CPM] and Standard Progressive Matrices [SPM] [24]; presence of eating disorders assessed through psychological assessment.

The study was conducted in accordance with the Declaration of Helsinki and was approved by the Ethics Committee of the Bambino Gesù Children’s Hospital [protocol code #2050/2020]. Written informed consent was obtained from the children’s parents or legal guardians. Children and adolescents provided written assent.

### Anthropometrics and biochemistry

Anthropometrics was evaluated using a Harpenden stadiometer to measure height and a calibrated scale to determine weight. Height, weight and BMI values were transformed into SDS according to Italian growth reference parameters [23].

Physical maturation was assessed by expert endocrinologists [AA, DF, MM, SC], who staged puberty according to Marshall and Tanner. Children were classified into Tanner stages based on breast development in girls and genitalia development in boys [25].

Participants underwent an OGTT [1.75 g/kg body weight up to a maximum of 75 g] with flavoured glucose [Glucosio Sclavo Diagnostics, 75 g/150 mL, Sovicille, Italy]. Blood glucose was assessed using the glucose oxidase technique [Cobas Integra, Roche, Indianapolis, IN, USA]; insulin levels were assessed using chemiluminescence on an ADVIA Centaur analyzer [Bayer Diagnostics, Siemens Healthcare, Erlangen, Germany]. Glycated haemoglobin [HbA1c] was tested using high-performance liquid chromatography [VARIANT II TURBO Haemoglobin Testing System; Bio Rad, Hercules, CA, USA].

### Investigative protocol

During the 1-day inpatient session, participants underwent three cognitive tasks in a fixed order [TS: Fluency tasks, Digit Span, and Non-Verbal Stroop] under both fasting [Fasting-Ts] and post-load [OGTT-Ts] conditions. Throughout the entire session, fNIRS monitored the PFC hemodynamic response function [HRF]. Data acquisition began before the OGTT, encompassing both the fasting condition resting state [Fasting-R] and the cognitive task execution [Fasting-Ts] immediately after the resting state condition. Specifically, the Fasting-R condition lasted 3 min, followed by approximately 20/30 min of Fasting-Ts. Following task completion, participants ingested the glucose load. Subsequently, 10 min after the glucose load, the OGTT-resting state condition began and lasted for 15 min; then, cognitive tasks [OGTT-Ts] were again administered. Time windows for OGTT-R and OGTT-Ts were determined based on previous fMRI studies that found greater cortical activation 15 min after glucose ingestion [26, 27]. Following the evidence from Xu et al. [26], we started fNIRS recording 15 min after the OGTT, beginning with the 15 min resting-state condition and immediately followed by the cognitive task condition at 31 min, with fNIRS recording interrupted approximately at min 60—70. This 60/70 min duration variability was driven by inter-individual differences in performance and engagement (especially brief pauses between cognitive tasks). The experimental paradigm was designed to minimize the patient’s discomfort and the risk of a drop in patient engagement with the study protocol after a series of demanding conditions [fNIRS setup montage, fasting, oral glucose administration, and blood sampling] that could easily cause children to drop out of the experiment.

The experimental procedure was conducted in a quiet and isolated room in order to reduce all environmental noise sources.

### Parameters of peripheral insulin metabolism

Insulin resistance [IR] was calculated using the HOMA [Homeostatic Model Assessment of Insulin Resistance] calculator provided by the University of Oxford [https://www.dtu.ox.ac.uk/homacalculator/], and the Insulin Sensitivity Index [ISI] was assessed according to Matsuda [28]. Insulin and glucose areas under the curve [AUC] were computed using the trapezoidal rule; insulin secretion index was estimated using the insulinogenic index [IGI] with IGI = [30 min insulin – fasting insulin [pmol/L]]/ [30 min glucose – fasting glucose [mmol/L]].

### Cognitive tasks

*Fluency tasks* were selected to evaluate verbal generative abilities and lexical access speed, which rely on cognitive flexibility and retrieval from long-term memory. In the Phonemic Fluency task [28], children were asked to verbalize as many words as possible beginning with a given phoneme [C, P and S]. The time limit was 1 min for each phoneme. Raw scores [number of correct words] were summed for each of the three trials, and the total z-scores based on the normative data were included in the analysis.

In the Semantic Fluency task [29], children were instructed to say as many words as possible in 1 min linked to a specific semantic category [colors, animals, fruits and cities].

Raw scores were summed and converted to Z-scores based on normative data, and total Z-scores were used in the statistical analysis.

*Digit Span* was selected as a measure of short-term and working memory from the Wechsler Intelligence Scale for Children-IV [30, 31]. Children must listen to a sequence of numbers and then repeat them in the same order or the reversed order [Backward Digit Span]. The sequence of numbers increases progressively with correct responses.

Raw scores were converted to scaled scores using normative data, and scaled scores were converted to z-scores for statistical analysis.

*Non-Verbal Stroop* from Leiter-3 [32, 33] was used to assess inhibitory control and cognitive flexibility, capturing the ability to suppress automatic responses and adapt to conflicting stimuli. Children had to identify a target among similar stimuli while inhibiting interference. The task included a congruent block, in which only one feature varied across stimuli, and an incongruent block, in which multiple features varied. Correct responses were counted for both congruent and incongruent blocks. Two measures were calculated: correct congruent responses [CCR] and correct incongruent responses [CIR]. For each measure, raw scores were converted to scaled scores based on normative data and into z-scores for the statistical analysis.

### Functional near-infrared spectroscopy

A 24-channel wearable continuous-wave fNIRS system [Brite24, Artinis Medical Systems B.V., Elst, The Netherlands] with dual-wavelength LED sources [760 and 850 nm] and 20 optodes [10 transmitters and 10 receivers] has been used. The system measures changes in optical density, which are converted into concentration changes in O_2_Hb [ΔO_2_Hb] and HHb [ΔHHb] using the modified Beer-Lambert law [19]. Enhanced brain activation induces locally increased blood flow, leading to an increase in O_2_Hb and a decrease in HHb concentration levels [34]. Participants were instrumented with a soft neoprene head cap [2.5 mm thickness, sizes S-L: S, 53–55 cm] placed over the frontal regions of the forehead. The probe consisted of 20 measurement points [source-detector distance of about 3 cm]. It was manually bilaterally placed over the frontal cortex, using an atlas-based approach based on Montreal Neurological Institute coordinates. Data was sampled with a frequency of 10 Hz and transmitted via Bluetooth to a laptop through Oxysoft software [Artinis Medical Systems, Elst, The Netherlands]. Based on different absorption spectra, PFC O_2_Hb and HHb concentration changes were calculated from changes in detected light intensity using the modified Lambert–Beer law, assuming a constant scattering [35]. fNIRS signals derived from 10 channels of each hemisphere were clustered into four areas: frontal pole [FP—right: S6D6, S9D6; left: S1D2, S4D2], DLPFC [right: S6D5, S8D5, S8D6; left: S1D1, S3D1, S3D2], ventromedial prefrontal cortex [VMPFC—right: S8D8, S9D8; left: S3D4, S4D4], and frontotemporal cortex [right: S8D7, S10D7, S10D8; left: S3D3, S5D3, S5D4].

### fNIRS data pre-processing

fNIRS data were analyzed using Nirstorm, a Brainstorm plugin for fNIRS data analysis [36], which is documented and freely available for download online under the GNU General Public License [http://neuroimage.usc.edu/brainstorm]. The raw signal was visually investigated, and data were considered invalid and manually excluded if motion artefacts were visible. Then, an algorithm was used to systematically remove motion artefacts using the Temporal Derivative Distribution Repair method [37]. We converted the raw data into HRFs for O_2_Hb, HHb, and tHb using the Modified Beer-Lambert Law, applying the differential pathlength factor correction method to account for participants’ ages and averaging the whole signal across the entire recording [38]. The last pre-processing step was a band-pass filter [0.01—0.1] to remove physiological noise. Signal processing was carried out according to the recently published guidelines for the best practices for fNIRS publications [20]. Finally, a block-average across tasks was performed, flattening the signal over the 30-s baseline before each task. The PFC activation during the first min of task administration was considered across the OGTT.

### Sample size calculation and statistical analysis

#### Sample size calculation

The sample size was determined using an a priori power analysis conducted in G*Power [version 3.1.9.7, The G*Power Team, Düsseldorf, Germany] [39]. Assuming two-tailed t-tests, a medium effect size [Cohen’s d = 0.6], a significance level [α] of 0.05, and a power of 0.80, a total of 24 participants was required. We used independent t-tests to investigate whether children with 2-h plasma glucose ≥ 140 mg/dl showed differences in cortical activity compared to those with the lowest 2-h glucose levels. No significant differences were found.

#### Cognitive tasks' analysis

Results on cognitive tasks were compared between sessions in fasting and glucose load conditions through a 2 × 5 Repeated Measure ANOVA [RM-ANOVA], with Time [Fasting-Ts v*s* OGTT-Ts] and Task [Semantic Fluency, Phonemic Fluency, Digit Span, CCR, and CIR] as main factors.

Significant interactions were further explored using Tukey-HSD post hoc comparisons. Partial eta-squared [ƞp^2^] was used as a measure of effect size.

#### fNIRS statistical analysis

The PFC activation during the resting state was calculated before [Fasting-R] and after glucose intake [OGTT-R], and the difference between PFC activation before and after glucose intake [OGTT-R minus Fasting-R] was included in the analysis.

Similarly, the PFC activation during cognitive tasks was calculated before [Fasting-Ts] and after [OGTT-Ts] glucose intake and the difference between PFC activity before and after glucose intake [OGTT-Ts minus Fasting-Ts] was included in the analysis. The comparisons between PFC activity during Fasting and OGTT were examined using a continuous paired t-test with a Monte Carlo permutation correction for multiple comparisons, False Discovery Rate [FDR] across time and derivations.

To compare the Fasting Resting-state 3-min signal [averaged over a 1-min epoch] with the continuous 15-min resting-state signal during OGTT, the OGTT resting-state signal was segmented into five consecutive 3-min epochs. Each epoch was subsequently segmented and averaged into non-overlapping 1-min windows [P1-P5] to obtain temporally localized estimates while maintaining adequate signal stability. This segmentation allowed comparisons of resting-state conditions of different durations. Averaging within 1-min windows embedded in longer epochs allows identifying and capturing slow hemodynamic changes while preserving temporal sensitivity to progressive physiological changes across the OGTT. In addition, previous work suggests that stable resting-state measures can be obtained from acquisitions of approximately 2.5—7 min, supporting the use of short segments within longer recordings to balance temporal resolution and signal robustness [40]. Results were considered significant at a p-value < 0.005, corrected for FDR.

To examine whether the insulin sensitivity and anthropometric measures [ISI, HOMA-IR, Insulin and Glucose AUC, IGI, and Tanner Stage] were related to delta activities [OGTT minus Fasting] in PFC, Pearson’s correlation analyses were run [also including partial correlations with age, sex, BMI and Tanner stage as control variables]. Results were significant with a p-value corrected for multiple comparisons.

Pearson’s correlation analyses were run to examine whether significant cognitive changes [from 2 × 5 RM-ANOVA] between Fasting-Ts and OGTT-Ts were related to delta HRF [OGTT minus Fasting] in PFC. Only significant results were included in the Results section.

## Results

The 28 participants’ mean non-verbal intelligence quotient was 108.8 ± 10.4 [SD]. No participants had fasting glucose ≥ 100 mg/dl, while 8 participants had 2-h plasma glucose ≥ 140 mg/dl, but none had glucose values ≥ 200 mg/dl. No difference in cortical activation was observed between participants with 2-h glucose < 140 mg/dl and those with 2-h glucose ≥ 140 mg/dl [all p > 0.16]. Table 1 summarises participants’ anthropometrics and biochemical parameters.

**Table 1:** Patients’ sociodemographic, anthropometric, and biochemical parameters

Sample size [n]	28
Sex [M/F]	17/11
Pubertal stage	12 [42.8%]
Stage I	2 [7%]
Stage II	1 [3.5%]
Stage III	4 [14%]
Stage IV	9 [32%]
Stage V	12.19 ± 2.44
Body weight [kg]	76.45 ± 17.63
Height [cm]	152.83 ± 12.31
BMI [kg/m^2^]	32.42 ± 4.22
BMI z-score [SDS]	2.62 ± 0.59
Fasting glucose [mg/dl]	82.04 ± 5.42
Fasting insulin [UI/ml]	14.84 ± 9.57
Glycated hemoglobin [mmol/L]	34.04 ± 3.02
30-min glucose [mg/dl]	128.44 ± 27.75
1-h glucose [mg/dl]	123.69 ± 25.60
90-min glucose [mg/dl]	127.36 ± 22.55
2-h glucose [mg/dl]	125.26 ± 20.41
Insulinogenic index [pmol/µmol]	1.25 ± 1
Glucose AUC [mg/dl/min^−1^]	112.72
Insulin AUC [mg /ml/min^−1^]	87.72
Matsuda [µmol/kg/pM]	4.49 ± 3.86
HOMA-IR [dimensionless]	3.07 ± 1.93
HDL-C [mg/dL]	52.06 ± 10.09
Triglycerides [mg/dl]	93.9 ± 43

AUC: Area Under the Curve; BMI: Body Mass Index; F: Female; HDL-C: High-Density Lipoprotein Cholesterol; HOMA-IR: Homeostatic Model Assessment of Insulin Resistance; M: Male; SD: Standard Deviation. Data are expressed as count and percentage or as mean ± SD [Standard Deviation]

Regarding cognitive tasks, the 2 × 5 repeated-measures ANOVA revealed significant main effects of time, F [1, 27] = 21.45, p = 0.00008, ƞp^2^ = 0.442 and TASK, F [4, 108] = 23.6, p < 0.00001, ƞp^2^ = 0.466. A significant Time × Task interaction was observed, F [4, 108]  = 3.70, p = 0.007, ƞp^2^ = 0.120. Tukey-HSD post-hoc comparisons indicated significantly higher z-scores during OGTT-Ts compared to Fasting-Ts for both Phonemic Fluency Task [p = 0.0006] and correct congruent responses in the Non-Verbal Stroop [p = 0.01].

### fNIRS results

#### Resting state in fasting and post-load conditions, i.e. the effect of glucose load

Continuous paired t-tests with Monte Carlo permutation of cortical activity during the five consecutive 1-min-averaged time windows of OGTT-R [from P1 to P5] against the Fasting-R were run.

Table 2 shows O_2_Hb and tHb changes in the left and right DLPFC measurement points during Fasting-R and OGTT-R.

**Table 2:** O_2_Hb and tHb in DLPFC measurement points during fasting resting, and OGTT resting states at time periods P2, P3, P4 and P5

	Fasting-R	OGTT-R
P2	O_2_Hb Left DLPFC [S3D1] [1.5 ± 2.0] *10^–6^ mol/L tHb Left DLPFC [S3D1] [1.6 ± 2.0] *10^–6^ mol/L	O_2_Hb Left DLPFC [S3D1] ** [4.1 ± 9.9]*10^–6^ mol/L tHb Left DLPFC [S3D1] ** [4.8 ± 2.0]*10^–6^ mol/L
P3	tHb Right DLPFC [S8D5] [1.1 ± 2.1]*10^–6^ mol/L	tHb Right DLPFC [S8D5] ** [3.4 ± 8.7]*10^–6^ mol/L
P5	O_2_Hb Left DLPFC [S3D1] [1.5 ± 1.9]*10^–6^ mol/L tHb Right DLPFC [S8D5] [1.2 ± 2.1]*10^–6^ mol/L O_2_Hb Right FrontalPole [S9D6] [5.7 ± 0.7]*10^–7^ mol/L	O_2_Hb Left DLPFC [S3D1] ** [4.3 ± 1.1]*10^–6^ mol/L tHb Right DLPFC [S8D5] ** [3.1 ± 7.6]*10^–6^ mol/L O_2_Hb Right FrontalPole [S9D6] [1.7 ± 0.7]*10^–6^ mol/L

Data expressed as means ± standard deviation. DLPFC: Dorsolateral prefrontal cortex; O_2_Hb: oxygenated hemoglobin; R: resting; tHb: total hemoglobin. **Cortical activation significantly different in the OGTT resting-state

**Fig. 1:**
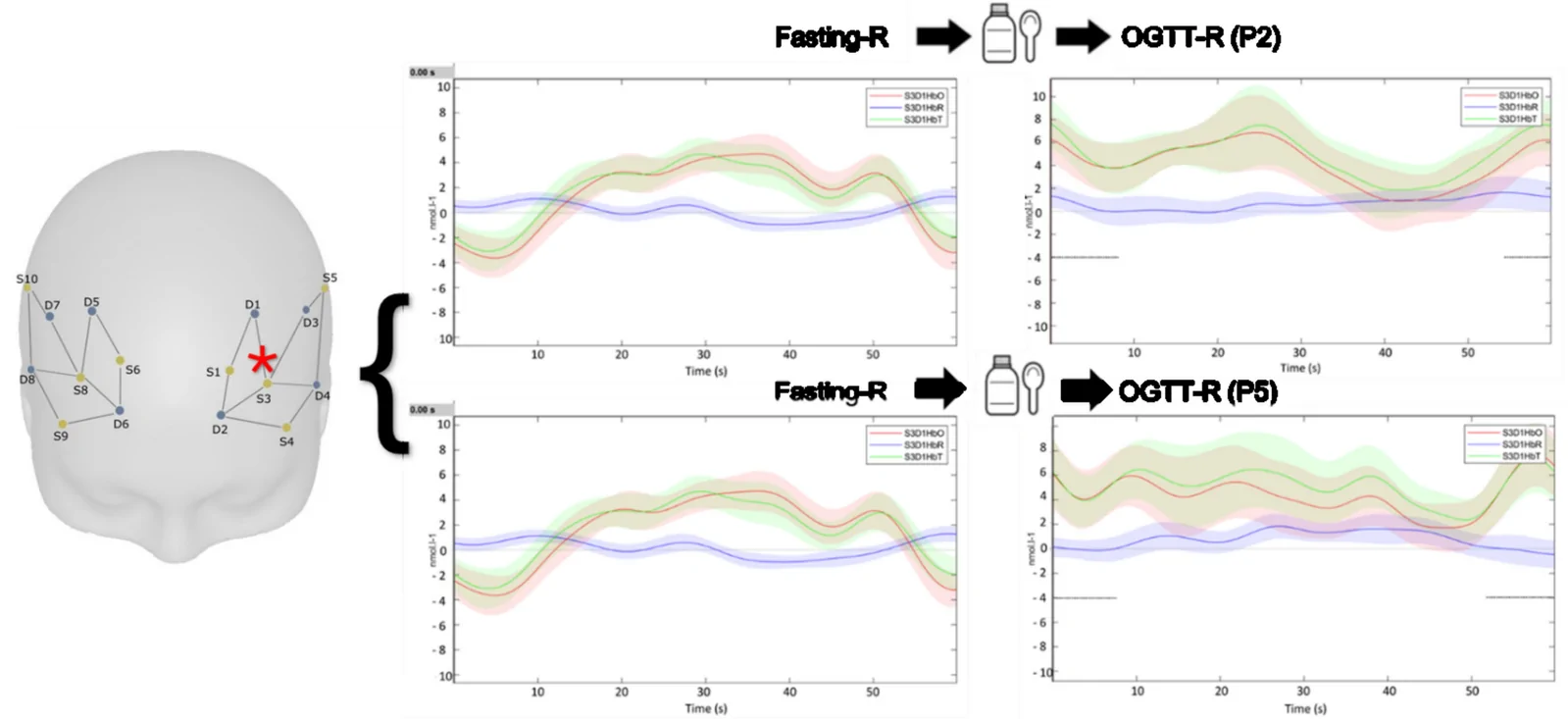
Left DLPFC [S3D1] hemodynamic response function (HFR) revealed as O_2_Hb [red line], HHb [blue line, and tHb [green line] concentration changes. Upper panels: significant difference between Fasting-R and OGTT-R in the second period [P2]. Lower panels: significant difference between Fasting-R state and OGTT-R in the fifth period [P5]. DLPFC: Dorsolateral prefrontal cortex; HHb; deoxygenated hemoglobin; O_2_Hb: oxygenated hemoglobin; R: resting; tHb: total hemoglobin. Shadows represent the standard deviation of HRF. See Methods section for details

At P2 [18—20 min after glucose load], both the O_2_Hb and tHb of the left DLPFC measurement point [S3D1] and the tHb of the right Frontal Pole measurement point [S9D6] showed greater activation [p-value = 0.0035, corrected for FDR] during OGTT-R as compared to Fasting-R. At P2, Pearson’s analysis showed a significant positive correlation between ISI and delta [post minus pre] activity at the left DLPFC measurement point [S3D1] for O_2_Hb [r = 0.61, p < 0.001] and tHb [r = 0.52. p = 0.005]. These results suggested that participants’ insulin sensitivity was significantly associated with greater activation in the left DLPFC during OGTT, with age, sex, BMI, and Tanner stage as control variables [partial correlation coefficient = 0.559, p = 0.004]. See Fig. 1 [upper panel] for hemodynamic differences at the left DLPFC measurement point [S3D1].

**Fig. 2:**
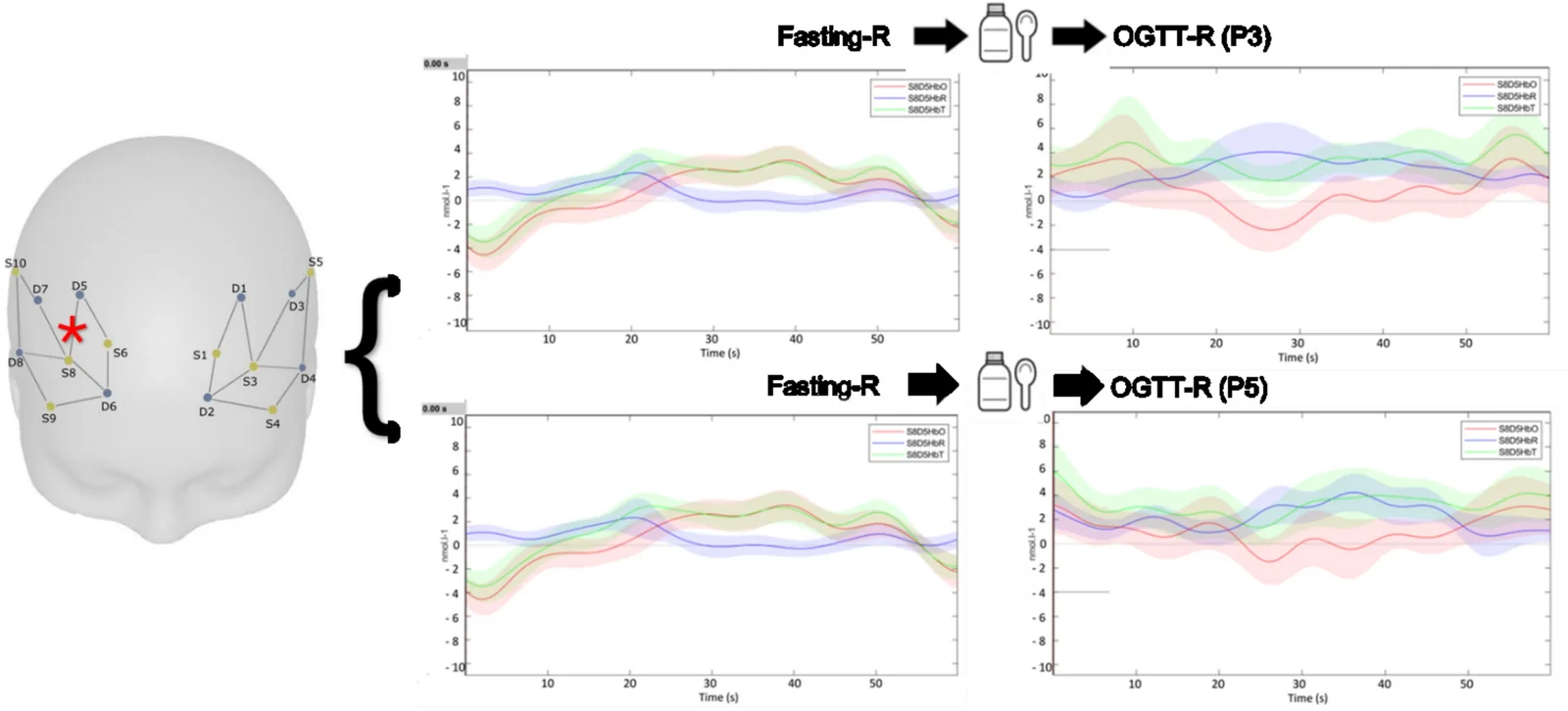
Left DLPFC [S8D5] hemodynamic response function (HFR) revealed as O_2_Hb [red line], HHb [blue line, and tHb [green line] concentration changes. Upper panels: significant difference between Fasting-R state and OGTT-R in the third period [P3]. Lower panels: significant difference between Fasting-R and OGTT-R in the fifth period [P5]. DLPFC: Dorsolateral prefrontal cortex; HHb; deoxygenated hemoglobin; O_2_Hb: oxygenated hemoglobin; R: resting; tHb: total hemoglobin. Shadows represent the standard deviation of HRF. See Methodssection for details

At P3 [21—23 min after glucose load], the tHb of the right DLPFC measurement point [S8D5] showed greater activation [p-value = 0.002, corrected for FDR] during the OGTT-R [see Fig. 2 – upper panel]. At P3, Pearson’s analysis showed a significant positive correlation between ISI and delta [post minus pre] activity at the right DLPFC measurement point [S8D5] for tHb [r = 0.73, p < 0.001]. These results suggested that participants’ insulin sensitivity was significantly associated with greater activation in the DLPFC during OGTT, considering age, sex, BMI and Tanner stage as control variables [partial correlation coefficient = 0.714, p = 0.0005].

Finally, at P5 [28—30 min after glucose load], the O_2_Hb of the left DLPFC measurement point [S3D1, p-value = 0.0033, corrected for FDR], the tHb of the right DLPFC channel [S8D5, p-value = 0.0029, corrected for FDR] and the tHb of the frontal pole measurement point [S9D6, p-value = 0.004, corrected for FDR] showed significantly greater activation during OGTT-R as compared to Fasting-R. Pearson’s analysis showed a significant positive correlation between ISI and delta [post minus pre] activity at the left DLPFC measurement point [S3D1] for O_2_Hb [r = 0.58, p = 0.001] and at the right DLPFC measurement point [S8D5] for tHb [r = 0.74, p < 0.001]. These results suggest that participants’ insulin sensitivity was significantly associated with greater activation in bilateral DLPFC during OGTT, considering age, sex, BMI and Tanner stage as control variables [S3D1: partial correlation coefficient = 0.511, p = 0.011; S8D5: partial correlation coefficient = 0.719, p = 0.0005].

See Figs. 1 and 2 [lower panel] for hemodynamic differences at the left and right DLPFC measurement points [S3D1 and S8D5].

#### Cognitive tasks in fasting and post-load conditions

In the Semantic Fluency task, the right fronto-temporal channel [S10D7] and right and left DLPFC channels [S3D3, S8D5 and S8D7] showed greater activation during OGTT-Ts as compared to Fasting-Ts for O_2_Hb [p-value < 0.005, corrected for FDR]. These differences were not associated with ISI, HOMA-IR or other estimates of insulin metabolism [p-value always > 0.1].

In the Digit Span task, we conducted separate analyzes for numbers repeated in the same order and for numbers repeated in reverse order. For the repetition of the number in the same order, significant differences were observed in the right DLPFC measurement point [S8D5] for tHb, suggesting greater activation during OGTT-Ts than during Fasting-Ts. For the repetition of the number in reverse order, significant differences were observed in the left DLPFC measurement point [S3D1] for HHb and the VMPFC measurement point [S3D4] for HHb, suggesting greater cerebral metabolic oxygen consumption for these measurement points at Fasting-Ts as compared to OGTT-Ts.

In the Non-verbal Stroop congruent block, significant differences were observed in the left frontal pole measurement point for tHb [S1D2], with greater activation during OGTT-Ts than during Fasting-Ts. A greater metabolic consumption at Fasting-Ts than OGTT-Ts was observed in the right frontal pole channel for HHb [S9D6].

In the non-verbal Stroop incongruent block, a greater cerebral metabolic oxygen consumption was observed in the left DLPFC channel for HHb [S3D1] at Fasting-Ts as compared to OGTT-Ts.

No other significant differences were found in cognitive tasks [p-value always > 0.1].

None of the differences observed during cognitive tasks in the Fasting-Ts and OGTT-Ts conditions was associated with ISI, HOMA-IR, or other estimates of insulin metabolism [p-values were always > 0.1].

## Discussion

Findings of our pilot study revealed enhanced cortical activation, as measured by O_2_Hb and tHb in the DLPFC, during the resting state following an OGTT in children with overweight or obesity. The extent of the hemodynamic responses was related to the degree of insulin sensitivity: the greater the activation of the bilateral DLPFC, the greater the peripheral Matsuda’s insulin sensitivity index. We focused on the relationship between the early cerebral response to an OGTT and the insulin-sensitivity in youths with overweight and obesity. Considering that most youths with obesity reach the glucose peak within 60 min after ingestion [41], and measures such as the Matsuda Index and AUCs at 30- and 60-min account for inter-individual differences, and consistent with the methodology of Xu et al. [26], we initiated fNIRS recordings at 15 min post-load to capture glucose-related cerebral effects.

Our results confirm the findings of a seminal positron emission tomography study that found glucose ingestion is associated with activation of PFC [8]. Children with higher peripheral insulin sensitivity exhibit a more glucose-responsive activation of the DLPFC, suggesting a greater capacity in insulin-sensitive children to inhibit food-seeking behaviours compared with insulin-resistant peers [18]. Investigating cerebral blood flow after glucose versus fructose intake in adolescents with either normal weight or obesity, Jastreboff et al. [18] found by fMRI reduced cerebral perfusion in the PFC in children with obesity following glucose load that did not covary with insulin or acyl-ghrelin. They concluded that this alteration may exert less inhibitory control, affecting motivation for food and eating behaviors. Notably, in our series we did not find any correlation with metabolic estimates referring to glucose and insulin dynamics during the first 30—60 min of the OGTT.

In the context of appetite control and food intake, the DLPFC plays a key role in regulating and inhibiting impulsive or unwanted eating behaviors, determining which foods are desirable or appropriate based on reward evaluation, individual goals and social context. Overall, the DLPFC integrates hunger, satiety, and reward signals from other brain regions involved in appetite control, [6].

Anthony et al. [42] showed, by positron emission tomography, that, while in healthy individuals, low circulating insulin levels enhance glucose brain metabolism in regions linked to appetite regulation and reward processing (such as ventral striatum and PFC), in insulin-resistant individuals, for the same insulin concentration, the glucose metabolism response is significantly attenuated. These findings align with our emerging results showing that insulin-sensitivity is associated with increased activity in the DLPFC.

There is uncertainty about the extent to which insulin reaches the central nervous system under various physiological conditions or during diagnostic and experimental procedures such as the OGTT and the hyperinsulinemic clamp.

By stimulating an endogenous insulin response, the OGTT better mimics real-life metabolic processes than the clamp and intranasal insulin administration.

A limitation of our current investigative approach is that we were unable to correlate the level of peripheral insulin resistance with brain insulin resistance, because we did not administer intranasal insulin [27].

Research using intranasal insulin and controlled stimulation showed that peripheral insulin resistance is associated with reduced central nervous system response, measured via fMRI, electroencephalography, or magnetoencephalography [8, 43]. At the molecular level, decreased insulin sensitivity, whether peripheral or central, involves lower receptor concentrations, reduced binding affinity, and disrupted signaling [40]. Insulin regulates blood flow by binding to endothelial receptors, increasing nitric oxide for vasodilation, but it can also trigger vasoconstriction via endothelin-1 and the mitogen-activated protein kinase pathway [44].

Consequently, insulin acts as a vasodilator at normal concentrations but as a vasoconstrictor at high concentrations [45]. Chronic hyperinsulinemia, associated with insulin resistance, promotes vasoconstriction and may become the driver of a loss of control in eating behaviour.

Significant task-related differences were observed, offering further insight into the interplay between glucose regulation and cortical activation in children with obesity. Increased O_2_Hb and tHb concentrations were detected following glucose intake across all tasks, without differences in behavioural performance or associations with insulin sensitivity. We found increased PFC activation during OGTT-TS compared with Fasting-TS. fNIRS likely captured an "online" learning process that was not behaviorally detectable. In line with this hypothesis, a study examining the neural basis of speech parsing found greater activation in the language-related areas after presenting previously presented word stimuli [46]. Similarly, our participants report that cognitive tasks feel more familiar after OGTT. Otherwise, this result may also reflect increased mental effort/workload.

It is essential to consider the difficulty of administering a combined fMRI and OGTT protocol on children and adolescents. fNIRS enabled us to examine the PFC hemodynamic response to the glucose load in an ecological context. Silent and resistant to movement artefacts, fNIRS enables continuous long-term measurements and offers a very high degree of experimental flexibility compared to other neuroimaging modalities. So far, thanks to its properties, wearability, versatility and motion tolerability, it has been used to predict food behaviours and to track responses to cognitive interventions [38]. We anticipated that fNIRS can also be a suitable tool for investigating the PFC response in children and adolescents, particularly those living with obesity and following lifestyle intervention and/or pharmacological treatment. In our study, high compliance towards fNIRS montage among participants, good signal quality, and no adverse events reported supported the implementation and applicability of this technique in pediatric populations. Childhood and adolescence mark a critical phase of brain development characterized by the progressive maturation of the PFC. Adolescents tend to gravitate towards calorie-dense foods, and excessive intake of these foods has the potential to disrupt self-regulatory processes by impacting brain function and behavioral control. These alterations may foster persistent maladaptive eating patterns. fNIRS has the potential to allow our understanding of the connections between adolescence, dietary choices, and brain function.

### Limitations and future perspectives

We acknowledge several limitations of the study. fNIRS recordings are inherently restricted to signals from the cortical surface, making it impossible to measure and evaluate activity in deeper brain areas that might also be associated with metabolism and cognition, limiting the interpretation of results. As said, we did not measure the response to intranasal insulin. The lack of a normal-weight control group makes it difficult to determine whether the observed patterns are unique to the population undergoing investigation or represent more general physiological mechanisms. The lack of a control experiment with a water load. The fixed sequence of cognitive tasks may have introduced bias. The sample size may limit generalizability and preclude adjustment for several covariates of results of this pilot. Investigating DLPFC activation via fNIRS after nutrient intake may provide insights into cognitive control of food intake. Results need to be replicated using other neuroimaging techniques, and further research is warranted to explore whether obesity treatment could modulate brain responses to glucose.

In conclusion, in the present study using fNIRS, we showed that insulin sensitivity in children and adolescents was correlated with greater activation of bilateral DLPFC after the OGTT, suggesting that peripheral insulin sensitivity reflects the PFC’s ability to control food-related behaviours. The association between insulin sensitivity and PFC activity might represent a protective mechanism in children to mitigate insulin-induced overeating.

## Supplementary Information

Below is the link to the electronic supplementary material.

Supplementary file1

## Data Availability

Some or all datasets generated during and/or analyzed during the current study are not publicly available but are available from the corresponding author on reasonable request.

## Abbreviations

AUC: Area under the curve
CCR: Correct congruent responses
CIR: Incongruent responses
DLPFC: Dorsolateral prefrontal cortex
fNIRS: Functional near-infrared spectroscopy
FP: Frontal pole
HHb: Deoxygenated hemoglobin
HOMA-IR: Homeostatic model assessment of insulin resistance
HRF: Hemodynamic response function
IGI: Insulinogenic index
ISI: Insulin sensitivity index
OGTT: Oral glucose tolerance test
OFC: Orbito frontal cortex
O_2_Hb: Oxygenated hemoglobin
PFC: Prefrontal cortex
tHb: Total hemoglobin
Ts: Tasks fasting-Ts and OGTT-Ts
VLPFC: Ventro-lateral prefrontal cortex
VMPFC: Ventromedial prefrontal cortex

## References

[1] Shashaj B, Luciano R, Contoli B, Morino GS, Spreghini MR, Rustico C, Sforza RW, Dallapiccola B, Manco M (2016) Reference ranges of HOMA-IR in normal-weight and obese young Caucasians. Acta Diabetol 53:251–260. 10.1007/s00592-015-0782-4 26070771 10.1007/s00592-015-0782-4

[2] Geisler CE, Hayes MR (2023) Metabolic hormone action in the VTA: reward-directed behavior and mechanistic insights. Physiol Behav 268:114236. 10.1016/j.physbeh.2023.114236 37178855 10.1016/j.physbeh.2023.114236 PMC10330780

[3] Leenaerts N, Jongen D, Ceccarini J, Van Oudenhove L, Vrieze E (2022) The neurobiological reward system and binge eating: a critical systematic review of neuroimaging studies. Int J Eat Disord 55:1421–1458. 10.1002/eat.23776 35841198 10.1002/eat.23776

[4] Stice E, Yokum S, Zald D, Dagher A (2011) Dopamine-based reward circuitry responsivity, genetics, and overeating. Curr Top Behav Neurosci 6:81–93. 10.1007/7854_2010_89 21243471 10.1007/7854_2010_89

[5] Kellar D, Craft S (2020) Brain insulin resistance in Alzheimer’s disease and related disorders: mechanisms and therapeutic approaches. Lancet Neurol 19:758–766. 10.1016/s1474-4422(20)30231-3 32730766 10.1016/S1474-4422(20)30231-3 PMC9661919

[6] Kullmann S, Heni M, Hallschmid M, Fritsche A, Preissl H, Häring HU (2016) Brain insulin resistance at the crossroads of metabolic and cognitive disorders in humans. Physiol Rev 96:1169–1209. 10.1152/physrev.00032.2015 27489306 10.1152/physrev.00032.2015

[7] Kleinridders A, Ferris HA, Cai W, Kahn CR (2014) Insulin action in brain regulates systemic metabolism and brain function. Diabetes 63:2232–2243. 10.2337/db14-0568 24931034 10.2337/db14-0568 PMC4066341

[8] Del Parigi A, Gautier JF, Chen K, Salbe AD, Ravussin E, Reiman E, Tataranni PA (2002) Neuroimaging and obesity: mapping the brain responses to hunger and satiation in humans using positron emission tomography. Ann N Y Acad Sci 967:389–397. 10.1111/j.1749-6632.2002.tb04294.x 12079866

[9] Batterink L, Yokum S, Stice E (2010) Body mass correlates inversely with inhibitory control in response to food among adolescent girls: an fMRI study. Neuroimage 52:1696–1703. 10.1016/j.neuroimage.2010.05.059 20510377 10.1016/j.neuroimage.2010.05.059 PMC2910204

[10] Le DS, Pannacciulli N, Chen K, Del Parigi A, Salbe AD, Reiman EM, Krakoff J (2006) Less activation of the left dorsolateral prefrontal cortex in response to a meal: a feature of obesity. Am J Clin Nutr 84:725–731. 10.1093/ajcn/84.4.725 17023697 10.1093/ajcn/84.4.725

[11] Reinert KR, Po’e EK, Barkin SL (2013) The relationship between executive function and obesity in children and adolescents: a systematic literature review. J Obes. 10.1155/2013/820956 23533726 10.1155/2013/820956 PMC3595670

[12] Gluck ME, Viswanath P, Stinson EJ (2017) Obesity, appetite, and the prefrontal cortex. Curr Obes Rep 6:380–388. 10.1007/s13679-017-0289-0 29071480 10.1007/s13679-017-0289-0

[13] Liang J, Matheson BE, Kaye WH, Boutelle KN (2014) Neurocognitive correlates of obesity and obesity-related behaviors in children and adolescents. Int J Obes (Lond) 38:494–506. 10.1038/ijo.2013.142 23913029 10.1038/ijo.2013.142 PMC4456183

[14] Riggs NR, Huh J, Chou CP, Spruijt-Metz D, Pentz MA (2012) Executive function and latent classes of childhood obesity risk. J Behav Med 35:642–650. 10.1007/s10865-011-9395-8 22218938 10.1007/s10865-011-9395-8

[15] Verbeken S, Braet C, Claus L, Nederkoorn C, Oosterlaan J (2009) Childhood obesity and impulsivity: an investigation with performance-based measures. Behav Change 26:153–167. 10.1007/s00787-007-0623-2

[16] Makaronidis JM, Batterham RL (2018) Obesity, body weight regulation and the brain: insights from fMRI. Br J Radiol 91:20170910. 10.1259/bjr.20170910 29365284 10.1259/bjr.20170910 PMC6223152

[17] Steward T, Miranda-Olivos R, Soriano-Mas C, Fernández-Aranda F (2019) Neuroendocrinological mechanisms underlying impulsive and compulsive behaviors in obesity: a narrative review of fMRI studies. Rev Endocr Metab Disord 20:263–272. 10.1007/s11154-019-09515-x 31654260 10.1007/s11154-019-09515-x

[18] Jastreboff AM, Sinha R, Arora J, Giannini C, Kubat J, Malik S, Van Name MA, Santoro N, Savoye M, Duran EJ, Pierpont B, Cline G, Constable RT, Sherwin RS, Caprio S (2016) Altered brain response to drinking glucose and fructose in obese adolescents. Diabetes 65:1929–1039. 10.2337/db15-1216 27207544 10.2337/db15-1216 PMC5384636

[19] Quaresima V, Ferrari M (2019) A mini-review on functional near-infrared spectroscopy (fNIRS): where do we stand, and where should we go? Photonics Basel 6(3):87. 10.3390/photonics6030087

[20] Cheng X, Stephen EP, Yücel MA (2026) Practical guide to statistical reporting in optical imaging. Neurophotonics 13(1):016601. 10.1117/1.NPh.13.1.016601 41821525 10.1117/1.NPh.13.1.016601 PMC12975399

[21] Val-Laillet D, Aarts E, Weber B, Ferrari M, Quaresima V, Stoeckel LE, Alonso-Alonso M, Audette M, Malbert CH, Stice E (2015) Neuroimaging and neuromodulation approaches to study eating behavior and prevent and treat eating disorders and obesity. Neuroimage Clin 8:1–31. 10.1016/j.nicl.2015.03.016 26110109 10.1016/j.nicl.2015.03.016 PMC4473270

[22] Pinti P, Tachtsidis I, Hamilton A, Hirsch J, Aichelburg C, Gilbert S, Burgess PW (2020) The present and future use of functional near-infrared spectroscopy (fNIRS) for cognitive neuroscience. Ann N Y Acad Sci 1464:5–29. 10.1111/nyas.13948 30085354 10.1111/nyas.13948 PMC6367070

[23] Cacciari E, Milani S, Balsamo A, Dammacco F, De Luca F, Chiarelli F, Pasquino AM, Tonini G, Vanelli M (2002) Italian cross-sectional growth charts for height, weight and BMI (6-20 y). Eur J Clin Nutr 56:171–180. 10.1038/sj.ejcn.1601314 11857051 10.1038/sj.ejcn.1601314

[24] Raven J (2008) The Raven progressive matrices tests: their theoretical basis and measurement model. In: Uses and abuses of intelligence studies advancing Spearman and Raven’s quest for (ed) non-arbitrary. Chapter 1 John Raven and Jean Raven Editors. Unionville New York Royal Fireworks. 17–68

[25] Tanner JM, Whitehouse RH (1976) Clinical longitudinal standards for height, weight, height velocity, weight velocity, and stages of puberty. Arch Dis Child 51:170–179. 10.1136/adc.51.3.170 952550 10.1136/adc.51.3.170 PMC1545912

[26] Xu F, Liu P, Pascual JM, Xiao G, Huang H, Lu H (2015) Acute effect of glucose on cerebral blood flow, blood oxygenation, and oxidative metabolism. Hum Brain Mapp 36:707–716. 10.1002/hbm.22658 25324201 10.1002/hbm.22658 PMC6869447

[27] Opstal AMV, Akintola AA, Elst MV, Westendorp RG, Pijl H, Heemst DV, Grond JV (2017) Effects of intranasal insulin application on the hypothalamic BOLD response to glucose ingestion. Sci Rep 7(1):13327. 10.1038/s41598-017-13818-x 29042645 10.1038/s41598-017-13818-x PMC5645424

[28] Matsuda M, DeFronzo RA (1999) Insulin sensitivity indices obtained from oral glucose tolerance testing: comparison with the euglycemic insulin clamp. Diabetes Care 22:1462–1470. 10.2337/diacare.22.9.1462 10480510 10.2337/diacare.22.9.1462

[29] Bisiacchi P, Cendron M, Gugliotta M, Tressoldi P, Vio C (2005) BVN 5–11 Batteria di valutazione neuropsicologica per l'età evolutiva. Erickson Trento Italy. **ISBN 9788879467193**

[30] Wechsler D (2003) Wechsler intelligence scale for children (4th Ed.). (WISC-IV): manual. The Psychological Corporation, San Antonio, TX, USA

[31] Orsini A, Pezzuti L, Picone L (2012) WISC-IV. Contributo alla taratura italiana. Giunti OS, Firenze

[32] Roid GH, Koch C (2017) Leiter-3 Nonverbal cognitive and neuropsychological assessment. Handbook of nonverbal assessment. In: McCallum R. (eds) Handbook of nonverbal assessment Springer, Cham.ì, Switzerland 10.1007/978-3-319-50604-3_8

[33] Cornoldi C, Giofrè D, Belacchi C (2016) Leiter-3 Leiter International Performance Scale Thirth Edition. Standardizzazione italiana. Giunti O.S., Firenze

[34] Ferrari M, Quaresima V (2012) A brief review on the history of human functional near-infrared spectroscopy (fNIRS) development and fields of application. Neuroimage 63:921–935. 10.1016/j.neuroimage.2012.03.049 22510258 10.1016/j.neuroimage.2012.03.049

[35] Huppert TJ, Diamond SG, Franceschini MA, Boas DA (2009) HomER: a review of time-series analysis methods for near-infrared spectroscopy of the brain. Appl Opt 48:D280–D298. 10.1364/ao.48.00d280 19340120 10.1364/ao.48.00d280 PMC2761652

[36] Tadel F, Baillet S, Mosher JC, Pantazis D, Leahy RM (2011) Brainstorm: a user-friendly application for MEG/EEG analysis. Comput Intell Neurosci 2011:879716. 10.1155/2011/879716 21584256 10.1155/2011/879716 PMC3090754

[37] Fishburn FA, Ludlum RS, Vaidya CJ, Medvedev AV (2019) Temporal derivative distribution repair (TDDR): A motion correction method for fNIRS. Neuroimage 184:171–179. 10.1016/j.neuroimage.2018.09.025 30217544 10.1016/j.neuroimage.2018.09.025 PMC6230489

[38] Scholkmann F, Wolf M (2013) General equation for the differential pathlength factor of the frontal human head depending on wavelength and age. J Biomed Opt 18(10):105004. 10.1117/1.jbo.18.10.105004 24121731 10.1117/1.JBO.18.10.105004

[39] Faul F, Erdfelder E, Lang AG, Buchner A (2007) G*Power 3: a flexible statistical power analysis program for the social, behavioral, and biomedical sciences. Behav Res Methods 39(2):175–191. 10.3758/bf03193146 17695343 10.3758/bf03193146

[40] Wang J, Dong Q, Niu H (2017) The minimum resting-state fNIRS imaging duration for accurate and stable mapping of brain connectivity network in children. Sci Rep 7(1):6461. 10.1038/s41598-017-06340-7 28743886 10.1038/s41598-017-06340-7 PMC5527110

[41] Nolfe G, Spreghini MR, Sforza RW, Morino G, Manco M (2012) Beyond the morphology of the glucose curve following an oral glucose tolerance test in obese youth. Eur J Endocrinol 166:107–114. 10.1530/eje-11-0827 22009494 10.1530/EJE-11-0827

[42] Anthony K, Reed LJ, Dunn JT, Bingham E, Hopkins D, Marsden PK, Amiel SA (2006) Attenuation of insulin-evoked responses in brain networks controlling appetite and reward in insulin resistance: the cerebral basis for impaired control of food intake in metabolic syndrome? Diabetes 55:2986–2992. 10.2337/db06-0376 17065334 10.2337/db06-0376

[43] Heni M, Kullmann S, Preissl H, Fritsche A, Häring HU (2015) Impaired insulin action in the human brain: causes and metabolic consequences. Nat Rev Endocrinol 11:701–711. 10.1038/nrendo.2015.173 26460339 10.1038/nrendo.2015.173

[44] Muniyappa R, Yavuz S (2013) Metabolic actions of angiotensin II and insulin: a microvascular endothelial balancing act. Mol Cell Endocrinol 378:59–69. 10.1016/j.mce.2012.05.017 22684034 10.1016/j.mce.2012.05.017 PMC3478427

[45] Fu J, Yu MG, Li Q, Park K, King GL (2021) Insulin’s actions on vascular tissues: physiological effects and pathophysiological contributions to vascular complications of diabetes. Mol Metab 52:101236. 10.1016/j.molmet.2021.101236 33878400 10.1016/j.molmet.2021.101236 PMC8513152

[46] McNealy K, Mazziotta JC, Dapretto M (2010) The neural basis of speech parsing in children and adults. Dev Sci 3:385–406 10.1111/j.1467-7687.2009.00895.x PMC3229831 20136936

