# Supplementary material for: fNIRS estimates prefrontal cortex activation following glucose load in youths with obesity
Source: J Endocrinol Invest. 2026 May 26;49(9):2497–507. doi: 10.1007/s40618-026-02911-6 (PMC13498646; doi:10.1007/s40618-026-02911-6)
Supplement: Supplementary file 1 [file 40618_2026_2911_MOESM1_ESM.pdf]

Supplementary Material

Supplementary Table 1. Cognitive tasks' scores in F and OGTT conditions

	F-Ts		OGTT-Ts		
	Mean ± SD		Mean ± SD		
Semantic Fluency task	-0.46	± 1.16	-0.44	± 1.13	
Phonemic Fluency task	-1.05	± 0.89	-0.37	± 1.04	***
Digit Span	-0.62	± 0.79	-0.49	± 0.83	
Non-Verbal Stroop CCR	0.45	± 0.92	1.00	± 1.11	*
Non-Verbal Stroop CIR	0.88	± 0.88	1.06	± 1.07	

Data are expressed as mean ± SD *(Standard Deviation); CCR: Correct Congruent Response; CIR: Correct Incongruent Response; * p < 0.05; *** p < 001*

Supplementary Table 2. Significant differences between cognitive tasks at fasting and after OGTT

	F-Ts	OGTT-Ts
Semantic Fluency task	**O_2Hb Left DLPFC (S3D3)** $(-6.0 \pm 9.9) * 10^{-6}$ mol/L	**O_2Hb Left DLPFC (S3D3)** ** $(-1.5 \pm 9.9) * 10^{-6}$ mol/L
	**tHb Left DLPFC (S3D3)** $(-5.5 \pm 9.8) * 10^{-6}$ mol/L	**tHb Left DLPFC (S3D3)** ** $(-1.6 \pm 8.3) * 10^{-6}$ mol/L
	**O_2Hb Right DLPFC (S8D5)** $(-2.9 \pm 6.2) * 10^{-6}$ mol/L	**O_2Hb Right DLPFC (S8D5)** ** $(-2.5 \pm 5.4) * 10^{-7}$ mol/L
	**O_2Hb Right Ventro-medial PFC (S8D7)** $(-6.0 \pm 1.5) * 10^{-6}$ mol/L	**O_2Hb Right Ventro-medial PFC (S8D7)** ** $(1.2 \pm 7.6) * 10^{-6}$ mol/L

	**O_2Hb Right fronto- temporal cortex (S10D7)** $(-1.5 \pm 2.1) * 10^{-6}$ mol/L	**O_2Hb Right fronto- temporal cortex (S10D7)** ** $(-4.2 \pm 3.7) * 10^{-6}$ mol/L
**Backward Digit Span**	**HHb left DLPFC (S3D1)** $(2.1 \pm 2.7) * 10^{-7}$ mol/L	**HbR left DLPFC (S3D1)** ** $(-1.3 \pm 3.4) * 10^{-7}$ mol/L
**Non-Verbal Stroop Congruent block**	**tHb Left Frontal Pole (S1D2)** $(-2.4 \pm 1.3) * 10^{-6}$ mol/L	**tHb Left Frontal Pole (S1D2)** ** $(2.7 \pm 8.1) * 10^{-6}$ mol/L
	**HbR Right Frontal Pole (S9D6)** $(6.8 \pm 8.2) * 10^{-7}$ mol/L	**HbR Right Frontal Pole (S9D6)** ** $(-3.1 \pm 7.4) * 10^{-7}$ mol/L
**Non-Verbal Stroop Incongruent block**	**HbR Left DLPFC (S3D1)** $(1.1 \pm 2.6) * 10^{-6}$ mol/L	**HbR Left DLPFC (S3D1)** ** $(-1.9 \pm 3.6) * 10^{-6}$ mol/L

*Data are expressed as mean ± SD (Standard Deviation). O_2Hb: Oxygenated Hemoglobin; DLPFC: Dorsolateral Prefrontal Cortex; tHb: Total Hemoglobin; HHb: Deoxygenated Hemoglobin; ** $p < 0.01$*

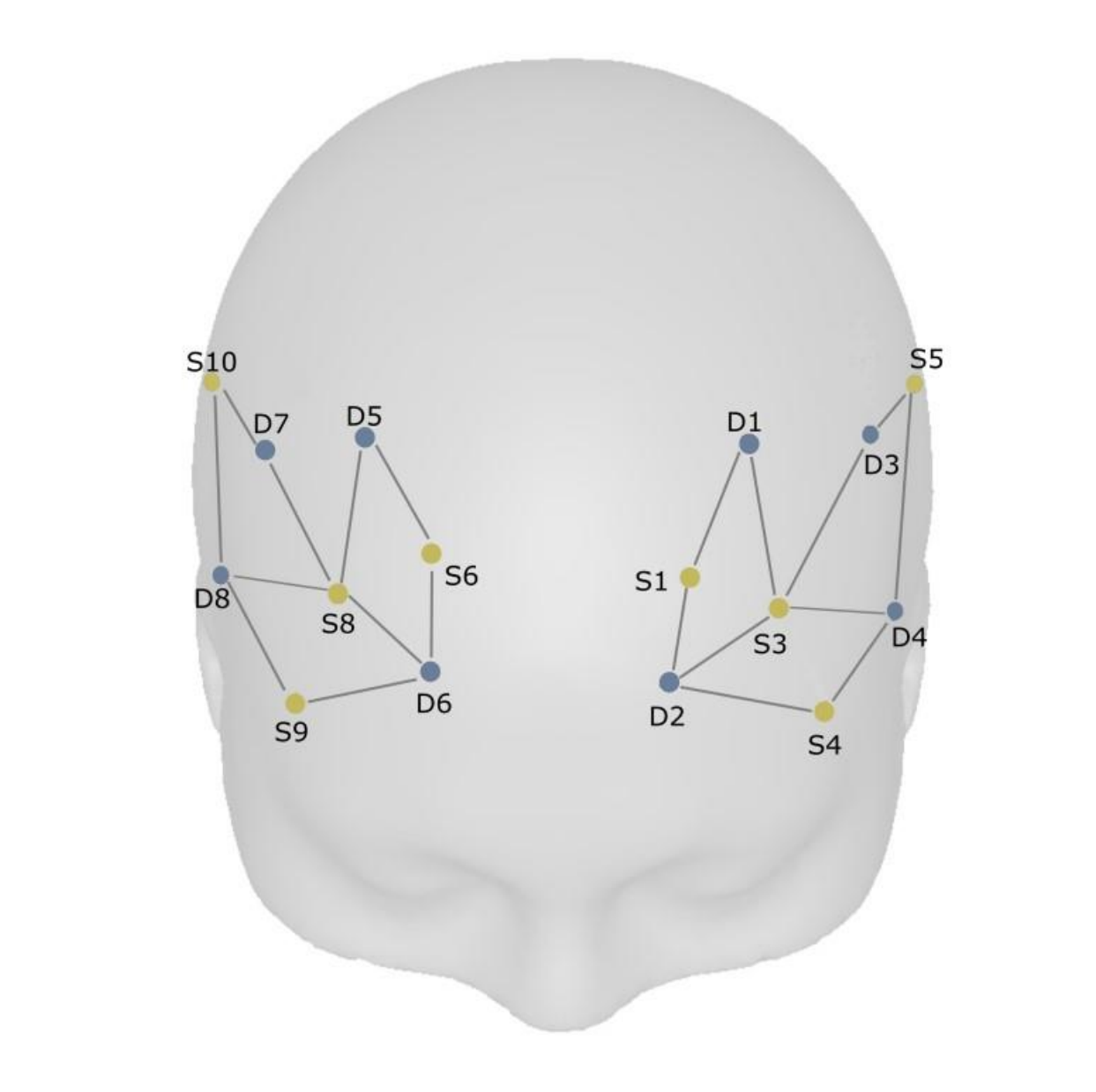

Supplementary Figure 1. *Placement of fNIRS optodes over the PFC. The 20 measurement points are represented. Yellow (S) and blue (D) circles represent the sources and detectors, respectively. The lines indicate the source-detector distance. See the Methods section for details.*

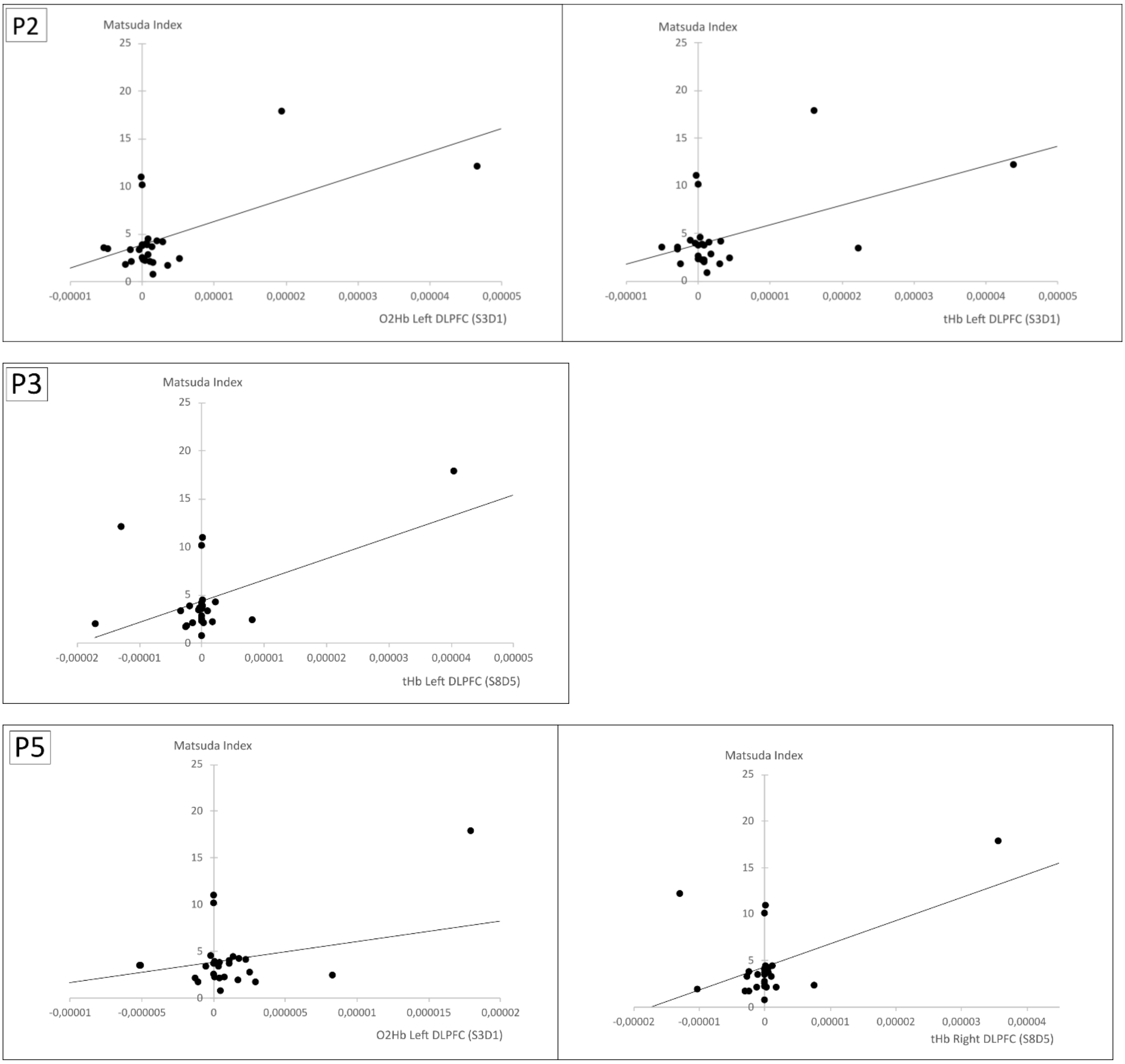

Supplementary Figure 2-4. Scatter plots of significant correlations. Respectively, P2 showed relationship between O_2Hb Left DLPFC (S3D1), tHb Left DLPFC (S3D1) and ISI (y axis). P3 showed relationship between tHb Right DLPFC (S8D5) and ISI (y axis). P5 showed relationship between O_2Hb Left DLPFC (S3D1), tHb Right DLPFC (S8D5) and ISI (y axis)
